# Therapeutic Assessment of Diverse Doxycycline-Based Formulations in Promoting Deep Corneal Wound Healing: Evidence from a Rat Model

**DOI:** 10.3390/vetsci12020143

**Published:** 2025-02-08

**Authors:** Sze-Min Chan, Ching-Li Tseng, Wei-Hsiang Huang, Chung-Tien Lin

**Affiliations:** 1 Graduate Institute of Veterinary Medicine, School of Veterinary Medicine, College of Bio-Resources and Agriculture, National Taiwan University, Taipei 106, Taiwan; d06629002@ntu.edu.tw; 2 Institute of Veterinary Clinical Sciences, School of Veterinary Medicine, College of Bio-Resources and Agriculture, National Taiwan University, Taipei 106, Taiwan; 3 Graduate Institute of Biomedical Materials and Tissue Engineering, College of Biomedical Engineering, Taipei Medical University, Taipei 110, Taiwan; 4 Graduate Institute of Molecular and Comparative Pathobiology, School of Veterinary Medicine, College of Bio-Resources and Agriculture, National Taiwan University, Taipei 106, Taiwan; 5 Department of Ophthalmology, National Taiwan University Veterinary Hospital, College of Bio-Resources and Agriculture, National Taiwan University, Taipei 106, Taiwan

**Keywords:** doxycycline-loaded gelatin nanoparticles, corneal stromal wounds, matrix metalloproteinase, rat model

## Abstract

Doxycycline (Dxy), a broad-spectrum antibiotic with anti-inflammatory effects, is commonly used in ophthalmology but is unstable as a topical eyedrop, degrading quickly into inactive forms and requiring frequent application. To address this, gelatin nanoparticles (GNPs) loaded with Dxy (DNPs) were developed as a stable ophthalmic nanomedicine for enhancing corneal wound healing by inhibiting matrix metalloproteinases (MMPs). In this study, female Sprague–Dawley rats underwent lamellar keratectomy, and various Dxy formulations—oral, conventional eyedrops, and DNP-containing eyedrops—were evaluated for corneal wound repair. Clinical assessments included fluorescein staining, slit-lamp biomicroscopy, spectral-domain optical coherence tomography (SD-OCT) imaging, histopathology, and immunohistochemistry for MMP-2, MMP-9, and α-SMA. The DNP group (0.01% Dxy in DNPs, applied twice daily) demonstrated faster corneal thickness recovery and epithelial healing on days 7 and 14 compared to 0.1% Dxy eyedrop treatments applied twice or four times daily. DNP-treated eyes also showed reduced angiogenesis intensity and lower MMP-2 and MMP-9 immunoreactive scores, with enhanced stromal recovery and reduced neovascularization. These results highlight DNPs’ potential as a superior treatment for corneal wounds, providing effective healing with less frequent dosing and lower drug concentrations. This study supports DNPs’ potential for clinical application as a stable and efficient therapeutic agent in ophthalmology.

## Simple Summary

This study evaluates doxycycline-loaded gelatin nanoparticles (DNPs) as an improved treatment for corneal wound healing by inhibiting matrix metalloproteinase (MMPs) in the cornea. Traditional topical doxycycline degrades quickly and requires frequent application, whereas DNPs offer a more stable formulation. A deep corneal wound model was established in rats. The effectiveness of treatment modalities was evaluated and compared between five groups, namely, no treatment (NC), DNP (0.01% doxycycline-loaded gelatin nanoparticles), topical doxycycline (0.1% at two different frequencies), and oral doxycycline. Clinical evaluations included fluorescein staining, slit-lamp biomicroscopy, optical coherence tomography (SD-OCT); histopathology, and immunohistochemistry with MMP-2, MMP-9, and myofibroblast markers(α-SMA) were done. Results showed that the DNP group achieved faster corneal thickness recovery and early epithelial healing within 14 days. The DNPs also reduced angiogenesis intensity by around 40% and lowered the immunoreactive scores for MMP-2 and MMP-9. Additionally, α-SMA cell levels in the DNP group were higher in the second week, indicating active tissue remodeling and reduced inflammation. Overall, the DNP-treated corneas exhibited faster recovery, minimal corneal neovascularization, and better visual acuity potential, requiring a lower concentration and application frequency. DNPs proved more effective in promoting corneal wound healing compared to traditional topical and oral doxycycline treatments.

## 1. Introduction

Corneal wound or corneal ulcerative disease is the damage to the corneal epithelium and stromal extracellular matrix (ECM) that leads to abrasions or ulcers. O’Neill et al. (2017) found that the prevalence of corneal ulcerative disease in dogs was 0.80% in England where most affected breeds were brachycephalic dogs [1]. Most superficial, uncomplicated corneal ulcers in animals heal effectively due to the highly regulated balance between corneal repair and degradation mechanisms. However, infected ulcers or those with significant stromal involvement can rapidly progress to corneal perforation within 24 h. This swift deterioration, clinically termed keratomalacia or corneal “melting”, is primarily driven by proteinases—enzymes derived from microbes, leukocytes, tear film, and corneal cells—that mediate stromal degeneration [2]. Under normal circumstances, healing is prompt and efficient when there is a superficial or uncomplicated injury to the cornea. During the initial injury that causes keratinocytes and stromal cell damage, as well as cell death, these incidents upregulated matrix metalloproteinase (MMP) expression [3,4,5]. The overexpression of MMPs delays cell migration from the limbus and corneal stromal remodeling, hence delaying wound healing; which causes the ulcerated cornea to succumb to further complications such as melting ulcers and secondary stromal infections [6,7,8].

Doxycycline (α-6 deoxy-5-hydroxytetracycline, Dxy), a member of the tetracycline antibiotic class, is a broad-spectrum antibiotic that prevents access of acyl t-RNA to the acceptor site on the mRNA-30s ribosomal subunit complex and a metal ion chelator [9,10]. Over the decades, it has also been found to have anti-inflammatory properties, making it useful in treating non-infectious ocular conditions such as rosacea and dry eye disease [11,12]. Therefore, not only can it inhibit MMP activity, but it also impedes the synthesis of these proteases by inhibiting the synthesis/secretion of the proinflammatory cytokine IL-1 [10,13,14]. Dxy as a topical eyedrop is unstable and tends to break down into epimers, which degrades its efficacy [15,16]. The quality of compounded doxycycline solution degrades by about 70% after 7 days of light-resistant storage at room temperature [17].

Topical ophthalmic drops can be washed out easily and require frequent application for constant tissue concentration. Pre-corneal fluid drainage is a key factor contributing to low ocular drug absorption [18]. Following instillation, approximately 80 to 90% of the administered volume is drained into the nasolacrimal duct [19]. Moreover, the epithelial layer of the cornea and conjunctiva serve as barriers to topical instillation of drugs [20]. Only <5% of an eyedrop-instilled dose can be delivered to the anterior eye after 5 min [21,22]. In recent decades, drug-loaded nanoparticles have drawn attention to new pharmaceutical formulation development. Nanoparticles are solid, colloidal particles consisting of macromolecular substances that vary in size from 10 nm to 1000 nm [23]. The advantages of nanoparticles as drug carriers applied in ophthalmology are increasing drug bioavailability and retention on/in ocular tissue with targeting capacity with safer, less invasive, and more effective options [20]. Varsha et al. revealed that as a topical ophthalmic drug delivery system, Dxy loaded in surfactant nanoparticles in a polymer gellan gum provides a better therapeutic strategy and less medicating frequency in the treatment of ocular bacterial infections [24]. A nanoparticle form of Dxy also demonstrated enhanced mucoadhesion, resulted in increasing pre-corneal retention time [25] with better cellular uptake by corneal epithelial cells and absorption [26,27].

This study uses gelatin, a natural biopolymer, as the matrix to form nanoparticles. Various drugs containing gelatin nanoparticles (GNPs) have been studied for treating dry eye syndrome and corneal neovascularization, showing their potential for enhancing ocular drug delivery efficiency [28,29]. GNPs have also proven successful in extending the residence time of the drug on the ocular surface [30].

In this study, a new formulation, DNPs (Dxy-loaded gelatin nanoparticles), was designed and synthesized. The purpose of this study was to evaluate the therapeutic effects of different doxycycline formulations: oral, eye drops, and nanomedicine (DNP)-containing eyedrops on treating deep stromal wounds via performing a lamellar keratectomy surgery in a rat model.

## 2. Materials and Methods

### 2.1. Doxycycline-Loaded Gel Nanoparticle (DNP) and Doxycycline Eyedrop Synthesis

The DNP was synthesized using a two-step desolvation method as described in previous studies [29,31]. In brief, type A gelatin derived from porcine skin, with a strength of 175 g (Sigma-Aldrich, St. Louis, MO, USA), underwent the first desolvation process to obtain a final concentration of 1% (*w*/*v*) purified gelatin. Doxycycline hyclate (Sigma-Aldrich, St. Louis, MO, USA), was dissolved in DMSO to acquire a 5 mg/mL concentration. Then, 50 µL of the doxycycline solution was added to 1 mL purified 1% gelatin under 800 rpm stirring speed. A 4 mL quantity of acetone was loaded into the gel mixture drop-wise using a peristaltic precision pump. A 50 µL quantity of 8% glutaraldehyde (Sigma-Aldrich, St. Louis, MO, USA) was then added to the mixture for cross-linking for 3 h. The mixture was transferred into a glass tube for rotatory evaporation (EYELA, Tokyo, Japan) to remove the organic solvent and obtain 1 mL of solution. The solution was centrifuged using an ultrafiltration tube (Vivaspin 6, 10 kDa MWCO) at 4000 rpm for 35 min. The unencapsulated Dxy was filtered out to the bottom solution and then discarded. The DNP concentrate was collected and redispersed with double-distilled water. The particle size and its zeta potential were measured by dynamic light scattering (DLS) (ZS90 Plus; Malvern, UK), operating at 25 °C, with light-scattering measurements taken at 90 degrees for 180 s. The particle structure was examined by a transmission electron microscope (TEM, Hitachi HT-7700, Tokyo, Japan) at an acceleration voltage of 75.0 kV. The DNP solution was dropped onto a carbon-coated nickel mesh and then tainted with 0.5% uranium acetate acyl (UA). High-performance liquid chromatography (HPLC) was employed to quantify the concentration of doxycycline. The HPLC configuration incorporated a LiChrospher^Ⓡ^ 100 RP-18 encapped reverse-phase column (5 μm) (Merk, Darmstadt, Germany), and the mobile phase was composed of water (containing 0.1% trifluoroacetic acid (TFA)), and acetonitrile (containing 0.1% TFA) in a volumetric ratio of 60:40; the mobile phase flowed at a rate of 1 mL/min detection using UV at 268 nm. Doxycycline encapsulation efficiency (E.E.) was determined by comparing the residual doxycycline content to the initially added amount, shown in percentage.

For the animal study, a doxycycline solution for eyedrops was prepared with 1 mg doxycycline hyclate dissolved in 1 mL phosphate buffer solution (PBS) (Sigma-Aldrich, St. Louis, MO, USA). A liquid preparation with a clear and light consistency was obtained. This colloidal solution was passed through 0.22 mm syringe filters for sterilization This eyedrop was freshly prepared every 5 days.

### 2.2. Animals and Deep Corneal Wound Rat Model

Female Sprague–Dawley rats, 6 to 8 weeks old, were used in this study. Animals were housed in standard cages with a controlled environment at a temperature of 20–25 °C under a 12 h light/dark cycle. The research protocol was approved by the Institutional Animal Care and Use Committee (IACUC) of the National Taiwan University (NTU-111-EL-00050).

A rat model of deep stromal ulcer was established by performing a lamellar keratectomy procedure on the right eye. The rats were anesthetized with a combination of Zoletil 20 mg/kg (Virbac, Carros, France) and xylazine 10 mg/kg (Balazine, Elite Bio-Science Inc., Taipei, Taiwan) via intraperitoneal injection. Alcaine 0.5% Ophthalmic Solution^®^ (Alcon, CA, USA) was applied topically to the right cornea as local anaesthesia. In brief, under the operating microscope, the surgical margin was defined with a 3 mm biopsy punch (Disposable Biopsy Punch, Integra, NJ, USA) gently rotating on the central corneal surface, creating the surgical outline. A beaver scalpel blade #64 (Miniature Blade Round-Tip Sharp 1 Side, Surgistar, CA, USA) was used to excise the epithelium and stroma material of the marked cornea area. Wound edges were trimmed with corneal scissors to acquire a circular margin. SD-OCT imaging of the surgical site was used to reference wound depth after the procedure to achieve a 50% excision of corneal thickness. Topical tobramycin (Tobrex^®^, Alcon, CA, USA) was applied as a prophylactic antibiotic (3 times per day for 7 days). Oral meloxicam (at 1 mg/kg) was given once daily for 5 days.

### 2.3. Treatment Regimen and Clinical Evaluation

Different Dxy formulations and their dosing frequencies are listed in Table 1. After stromal wound creation, 40 rats were divided into 5 groups tested with different agents: control with no treatment (NC); oral doxycycline (OD) 10 mg/kg, syringe fed 2 times per day; doxycycline topical eyedrop given 4 times per day (DXY4); doxycycline topical eyedrop given 2 times per day (DXY2); and doxycycline-loaded gelatin nanoparticles, 2 times per day (DNP). From each group, 2 rats were randomly selected to measure the contralateral eye to serve as the normal eyes (NORM). These 5 groups were further divided based on the duration into 7-day and 14-day subgroups, with each subgroup consisting of 4 rats.

Clinical evaluation was carried out prior to surgery as a baseline parameter. The evaluations included: slit-lamp biomicroscopy (KOWA SL-17 Portbale Slit-lamp, KOWA, Tokyo, Japan), 1% fluorescein dye staining, and spectral domain optical coherence tomography (SD-OCT) (Spectralis^®^, Heidelberg, Germany). Clinical assessment was performed immediately after surgical keratectomy on day 1, day 2, day 3, day 7, and day 14. Slit-lamp biomicroscopy was used mainly to determine any abnormalities on the ocular surface and the anterior uvea of the eyes. Fluorescein dye was used to detect disrupted cornea integrity. Photography was performed simultaneously as the images were analyzed using image analyzing software (Image J 1.38x; National Institutes of Health, Bethesda, MD, USA). SD-OCT was performed with the machine set to the anterior segment module to examine the cornea. The thickness of the epithelium and stromal layers was measured using OCT image analyzing software (Heidelberg Eye Explorer, Heidelberg Engineering, MA, USA). Three measurement points were made on each cornea: central (point-1); medial (point-2); and lateral (point-3), 1000 µm from the central cornea (please refer to Figure S2). The thickness was calculated as the average of measurements taken at these 3 distinct points.

### 2.4. Histopathology and Immunohistochemistry

On day 7 and day 14, the rats from each subgroup (*N* = 4) corresponding to the designated duration were euthanized for histopathology and immunohistochemistry evaluations of the cornea. The eyes were enucleated and fixed in 10% neutral buffered formalin for 24 h followed by vertically sectioning, dehydration processes, and paraffin embedding. A 4 µm sagittal section through the pupil and optic nerve was stained with hematoxylin-eosin (H&E), and the slides were examined under light microscopy. Immunohistochemistry of the cornea was performed based on previously reported methods [32]. The score was obtained by multiplying the percentage of positive cells (PP) by the primary staining intensity (SI) and assessed as follows: PP values: 0 (no positive cells), 1 (<25% positive cells), 2 (25–50% positive cells), 3 (51–75% positive cells), and 4 (>75% positive cells); SI values: 0 (negative), 1 (weak), 2 (moderate), and 3 (strong) [33]. The primary antibodies specifically targeting α-SMA (ab5694, Abcam, Waltham, MA, USA), MMP-2 (ab86607, Abcam, Waltham, MA, USA), and MMP-9 (ab76003, Abcam, Waltham, MA, USA) were used in this study.

### 2.5. Statistical Analysis

Statistical software IBM SPSS Statistics for Windows, version 29 (IBM Corp., Armonk, NY, USA) was used in this study. The data were tested normal The clinical findings from each group were tested with the mixed model repeated measure with a post hoc pairwise Bonferroni test. A one-way ANOVA test with post hoc pairwise Bonferroni test was carried out to analyze the treatment effects on different time points. The histopathology and immunohistochemistry analysis were tested with one-way ANOVA and post hoc pairwise Bonferroni tests.

## 3. Results

### 3.1. Characteristics of DNP

**Figure 1:**
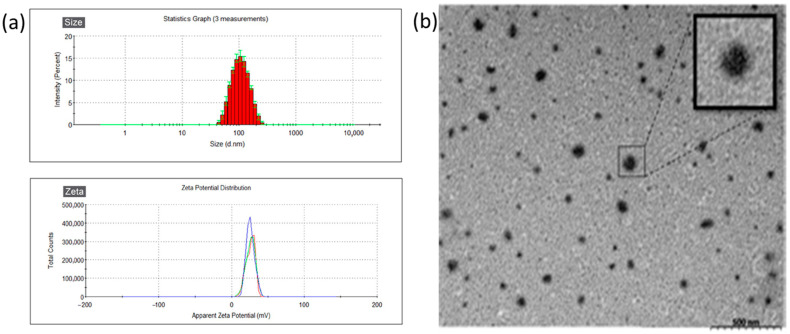
Characterization of DNPs. (**a**) The size distribution pattern, and Zeta potential peak for DNPs acquired by DLS analysis. (Triple repetition displaying in 3-colored line graph). (**b**) Particle image acquired. Data are presented as mean ± SD, n = 4.

The gelatin nanoparticles (GNPs) and Dxy-loaded GNPs (DNPs) were examined by DLS. The results of their size and zeta potential are shown in Table 2. The average particle size of GNP was 98.83 ± 0.68 nm. A bigger particle size (132.6 ± 0.2 nm) of DNP (the Dxy-loaded) was found. The zeta potential of these two nanoparticles was in positive condition at a similar range (21.5 mV and 22.4 mV) which can help them to adhere to the ocular surface (more negative condition). These nanoparticles revealed low polydispersity index (PDI) values (<0.2), and DNPs with a narrow size distribution are observed in Figure 1a. Round and spherical nanoparticles (DNPs) were observed from TEM images. The Dxy encapsulation efficiency in GNPs (DNPs) was 53.8% ± 4.25%, which was not high but acceptable.

### 3.2. Effects of Doxycycline on Clinical Findings

#### 3.2.1. Corneal Neovascularization

**Figure 2:**
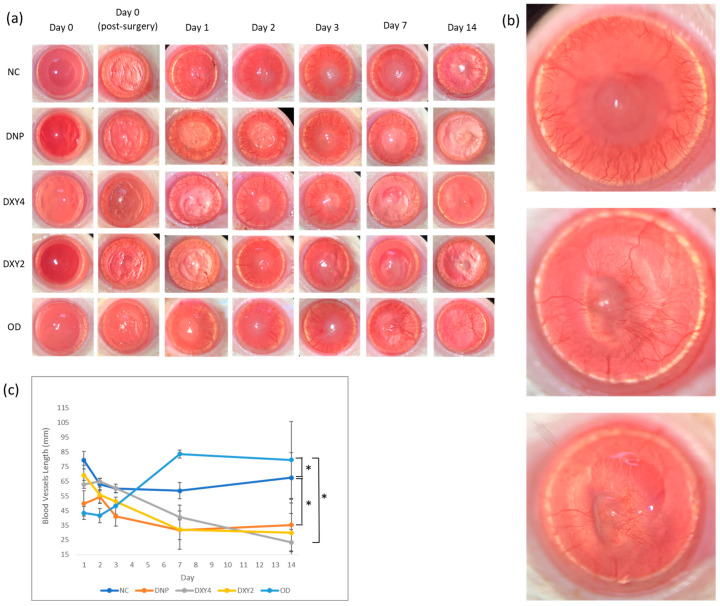
The effects of treatment on corneal neovascularization. (**a**) Photography images of corneal neovascularization progression of each group from day 0 to day 14. Presence of blood vessels on day 1 in all experimental groups. Each group developed noteworthy amounts of blood vessels during the first 3 days post-surgery. Note that the NC and OD group also showed corneal edema, especially at the central cornea region. By day 7, the corneal edema still persisted in the NC group; the OD group had long vascularization projecting across the radius of the cornea. By day 14, the presence of minimal and short vascularization was noted in groups DNP, DXY2, and DXY4; the NC and OD groups persistently showed longer projections of blood vessels, concentrated at the medial side of the cornea. (**b**) Magnified photography images of 3 representative cornea. Top to bottom: Control group day-1, control group day 7, control group day 14. (**c**) Corneal neovascularization length (mm) changes over the 14-day study. The neovascularization was first noted on day 1 post-surgery. The untreated group (NC) displayed a persistently high blood vessel length. Neovascularization was noted in all groups in the first 3 days, with the OD group showing delayed blood vessel formation, where it increased after day 3 post-surgery and persisted abundantly throughout the study. The treated groups (DNP, DXY2, and DXY4) demonstrated a trend of blood vessel reduction after day 2 which remained minimal throughout the study. The DNP group developed significantly fewer blood vessels throughout the study (* *p* < 0.005).

Every experimental subject developed corneal neovascularization on day 1 post-operatively. Photos of blood vessel development in the cornea are shown in Figure 2a, and the quantification of blood vessel length is revealed in Figure 2b. All the treated groups except the non-treatment (NC) and oral doxycycline group (OD) showed a trend of reduction over the 14 days of the study. The main effects of the groups were highly significant (F (4, 42.647) = 5.250, *p* = 0.002). Post hoc pairwise comparison showed that the DNP and OD group were significantly different from the NC group (*p* = 0.022, 95% C.I. = [1.585, 32.184] and *p* = 0.006, 95% C.I. = [3.894, 34.520], respectively).

The OD group demonstrated an increasing trend of having the lowest blood vessel count after the first 3 days. However, on days 7 and 14, their neovascularization development marked the highest blood vessel figure, showing that the oral medication lacked anti-angiogenic effects. The DXY4 group had significantly different main effects of corneal neovascularization compared to the OD group (*p* = 0.032, 95% C.I. = [0.841, 30.685]). Though the DXY4 group started with moderately high vascular development, the total length of blood vessels after day 3 regressed to the lowest among the groups by day 14 (Figure 2b). The DNP-treated group showed in lowest vessel length on day 3 (Figure 2b) and is still effective for inhibiting vessel formation on days 7 and 14. A similar tendency was also found in the DXY2 group (Figure 2b).

#### 3.2.2. Corneal Epithelial Wound Healing

**Figure 3:**
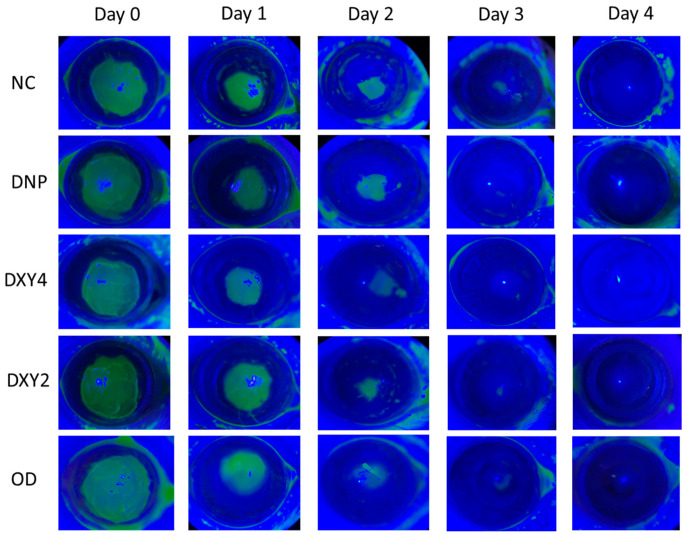
Photography images of corneal fluorescein staining of each group from day 0 to day 14. The DNP group did not stain by day 3 compared to other doxycycline-treated groups, which still marked a minuscule stain. All subjects did not stain positive by day 4 (*p* = 0.084).

Lamellar keratectomy surgery was performed on day 0, and the progress of corneal wound healing was observed starting day 1 post-surgery. Figure 3 shows the gross images of corneal fluorescein staining indicating the wound healing rate. On day 1, the fluorescein-stained areas in the DXY4 and DNP groups were smaller than those in the other groups, showing less cornea damage with an accelerated healing rate. Though the NC and OD groups showed a slower healing rate by day 3, there were no significant differences in the healing rate among the groups after 4 days (*p* = 0.084).

#### 3.2.3. Corneal Epithelial and Stromal Thickness

**Figure 4:**
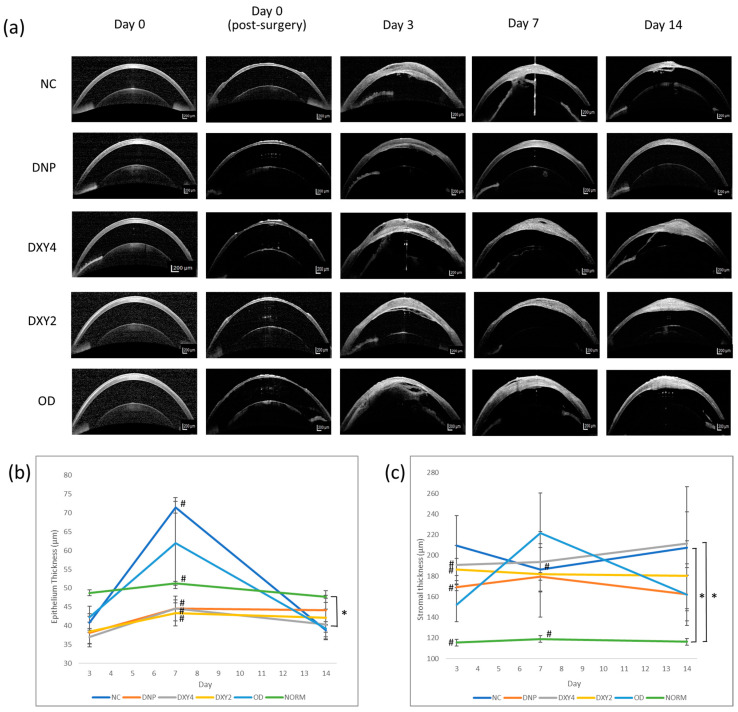
Corneal epithelium and stromal thickness analysis over 14 days. (**a**) OCT images of the cornea. Comparison of the epithelium and stromal thickness between groups prior to surgery (normal), immediately after lamellar keratectomy surgery(Day 0 post-surgery), and days 3, 7, and 14 post-surgery. The DNP group had less severe stromal thickening throughout the study and more homogenous stromal and epithelial cell proliferation. (**b**) Epithelium thickness (µm) between groups. * *p* < 0.05 indicates a significant difference between NORM and DXY4. # *p* < 0.05 indicates a significant difference between NC and DNP, DXY4, DXY2, and NORM. (**c**) Stromal thickness (µm) between groups. * *p* < 0.05 indicates a significant difference between groups. # *p* < 0.05 indicates a significant difference between groups by days 3 and 7.

We compared the epithelium and stromal thickness to the normal rats (NORM group) to justify the treatment effects of each group’s epithelial and stromal cell growth. Figure 4 shows the mean ± SEM of corneal epithelium and stromal thickness over time. Corneal epithelial thickness was tested to have significant main effects in the groups (F (5, 44.113) = 2.685, *p* = 0.033). In the post hoc pairwise comparison, the DXY4 group significantly differed from the NORM group (*p* = 0.036, 95% C.I. = [−18.198, 0.356]). The other groups did not show epithelial thickness differences throughout the study. In general, all the subjects had regained their epithelium layer by day 3 post-surgery. The NC and DO group had prominent epithelial thickening by day 7 but regressed to moderate thickness by day 14. ANOVA was performed on days 3, 7, and 14, respectively, to identify the group differences on these days. On day 7, there was statistical significance in the difference between groups. A post hoc Games–Howell test revealed that the thickness of the epithelium of the NC group was significantly thicker than the DNP (*p* < 0.001, 95% C.I. = [19.599, 34.162]), DXY4 (*p* = 0.001, 95% C.I. = [13.186, 40.591]), DXY2 (*p* = 0.003, 95% C.I. = [12.678, 42.522]), and normal group (*p* < 0.001, 95% C.I. = [13.694, 26.805]).

**Figure 5:**
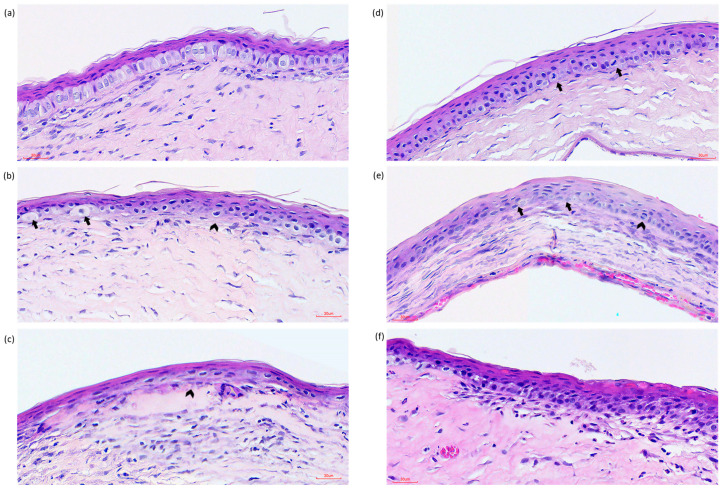
Corneal epithelium findings on day 7. (**a**) DNP group. Single-layer, well-organized basal cells and presence of intact EBM. (**b**) NC group. Haphazard basal cells, vacuolation (arrow), and absence of EBM (arrowhead). (**c**) NC group. Absence of basal cells and EBM, presence of thin layers of stratified keratocytes. (**d**) DXY 4 group. Presence of mitotic figures (arrow) and hyperplastic wing cells. (**e**) DXY2 group. Areas with absence of EBM (arrow), areas with mitotic figures and haphazard basal cells and wing cells (arrowhead). (**f**) OD group. Haphazard basal cells with hyperplastic layers of basal and wing cells and absence of EBM.

When we examined the morphology of the epithelium and stroma via histopathology, only the DXY2 and DXY4 groups sporadically exhibited mitotic figures at the basal cell layer of the epithelium (Figure 5). By day 7, we observed that rats that were treated with DNPs had restoration of the single-layer basal cells of the epithelium and the presence of epithelium basement membrane (EBM), whereas the NC and OD groups had more layers of stratified superficial cells with basal cell layers that were haphazardly arranged, with the presence of more wing cells and a lack of EBM.

The stromal thickness was the significant main effect of the groups (F (5, 41.746) = 4.004, *p* = 0.005) (Figure 4). Overall, all the experimental groups had thicker stromal layers than the normal rats throughout the study after the surgical procedure. However, only the NC and DXY4 groups were significantly thicker than the NORM group (*p* = 0.004, 95% C.I. = [19.808, 156.498] and *p* = 0.012, 95% C.I. = [11.387, 148.077], respectively) (Figure 4). The DNP group had the least stromal thickening throughout the study. ANOVA was applied separately to test for stromal thickness difference between groups on days 3, 7, and 14. By day 3 of treatment, the three topical eyedrop-treated groups (DNP, DXY4, and DXY2) had significantly thicker stromal layers than the normal group (DNP: *p* = 0.032, 95% C.I. = [−103.057, −4.076], DXY4: *p* = 0.042, 95% C.I. = [−147.739, −2.594], DXY2: *p* = 0.003, 95% C.I. = [−113.264, −28.307]).

### 3.3. Histopathological Findings

#### 3.3.1. Effects of Doxycycline on the Expression of MMP-2 and MMP-9 and Neutrophil Infiltration

**Figure 6:**
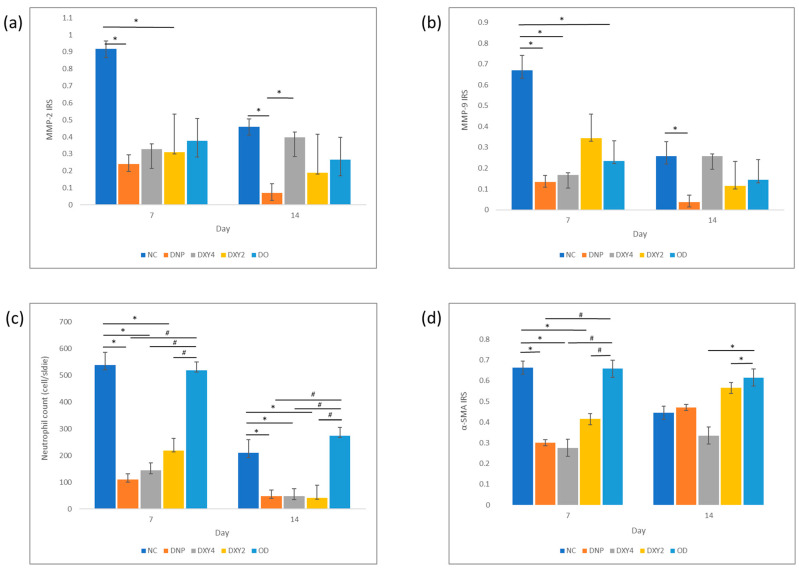
Immunoreactivity scores (IRS) of MMP-2, MMP-9, and α-SMA immunohistochemistry staining and neutrophil count with Hematoxylin and Eosin (HE) staining analysis. (**a**) MMP-2 IRS. By day 7, the NC group scored significantly higher than the DNP and DXY2 groups (* *p* < 0.05). By day 14, the DNP group scored significantly lower than the NC and DXY4 groups (* *p* < 0.05). (**b**) MMP-9 IRS. By day 7, the NC group scored significantly higher than all the treatment groups except the DXY2 group. By day 14, the DNP and NC groups showed a significant difference (* *p* < 0.05). (**c**) Neutrophil cell counts were measured by cell counting per slide. On both days 7 and 14, * *p* < 0.05 and # *p* < 0.05 indicated a significant difference between the NC and OD groups compared to the other 3 topically treated groups. (**d**) α-SMA IRS. By day 7, * *p* < 0.05 and # *p* < 0.05 indicated a significant difference between the NC and OD groups and the other 3 topically treated groups. By day 14, the OD group scored significantly higher than DXY2 and DXY4 (* *p* < 0.05).

**Figure 7:**
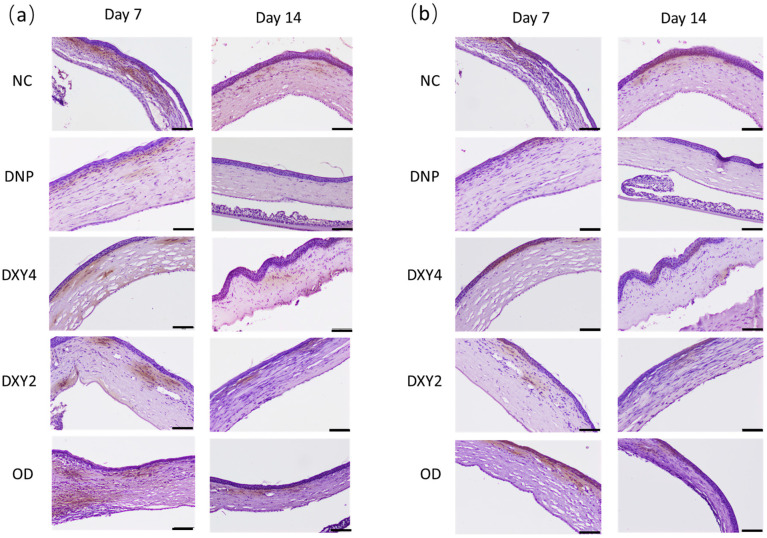
Immunohistochemistry staining with MMP-2 and MMP-9 markers at days 7 and 14. (**a**) MMP-2 immunohistochemistry. By day 7, the NC and OD groups were heavily stained. The DNP, DXY4, and DXY2 groups expressed MMP-2 staining mainly at the anterior part of the stroma. (**b**) MMP-9 immunohistochemistry. By day 7, the expression of MMP-9 stains was mainly at the anterior stroma, immediately beneath the EBM. The DNP and DXY4 groups had minimal staining by day 14. Scale bars: 100 µm.

A one-way ANOVA was performed to compare the effect of different treatments on the expression of MMP-2 and MMP-9 markers in the cornea. The test revealed a statistically significant difference in MMP-2 expression between at least two groups (F (4, 11) = 4.532, *p* = 0.021) on day 7 (Figure 6 and Figure 7). Tukey’s HSD Test for multiple comparisons found that the mean value of the MMP-2 IRS of the NCgroup was significantly different from that of the DNP group and DXY2 group (*p* = 0.023, 95% C.I. = [0.087, 1.267] and *p* = 0.043, 95% C.I.= [0.017, 1.091], respectively). There was no statistically significant difference between other groups (*p* > 0.05), though generally, all the treatment groups had much less IRS than the NC group. By day 14, there was a statistical difference between groups (F (4, 10) = 4.878, *p* = 0.019). A post hoc Tukey’s HSD test demonstrated that the NC group and DXY4 group still showed significantly higher IRS compared to the DNP group (*p* = 0.019, 95% C.I.= [0.064, 0.717] and *p* = 0.050, 95% C.I.= [0.000, 0.653], respectively). Rats treated with doxycycline gel-loaded nanoparticles showed the lowest MMP-2 IRS after 14 days of treatment.

MMP-9 immunomarker expression showed a similar expression trend with an overall lower intensity. There was a statistical difference between groups on both day 7 and day 14, (F (4, 11) = 6.748, *p* = 0.005) and (F (4, 10) = 7.641, *p*= 0.004), respectively. After 7 days of treatment, all the treated groups had much lower MMP9 immuno-expression compared to the non-treated NC group. A post hoc Tukey’s HSD test demonstrated that the NC group had significantly higher MMP-9 than the DNP (*p* = 0.006, 95% C.I. = [0.153, 0.920]), DXY4 (*p* = 0.10, 95% C.I.= [0.120, 0.887]) and OD groups (*p* = 0.016, 95% C.I. = [0.076, 0.793]). A post hoc Tukey’s HSD on day 14 showed that the DNP group had significantly less MMP-9 IRS than the NC and DXY4 groups ((*p* = 0.007, 95% C.I. = [0.061, 0.377]) and (*p* = 0.007, 95% C.I. = [0.061, 0.377]), respectively). Both MMP-2 and MMP-9 immunostaining shared a similar score on these 2 days. Overall, IRS had decreased prominently after 14 days of study. The DNP group had the lowest IRS regardless of the type of MMP or the day of the study.

**Figure 8:**
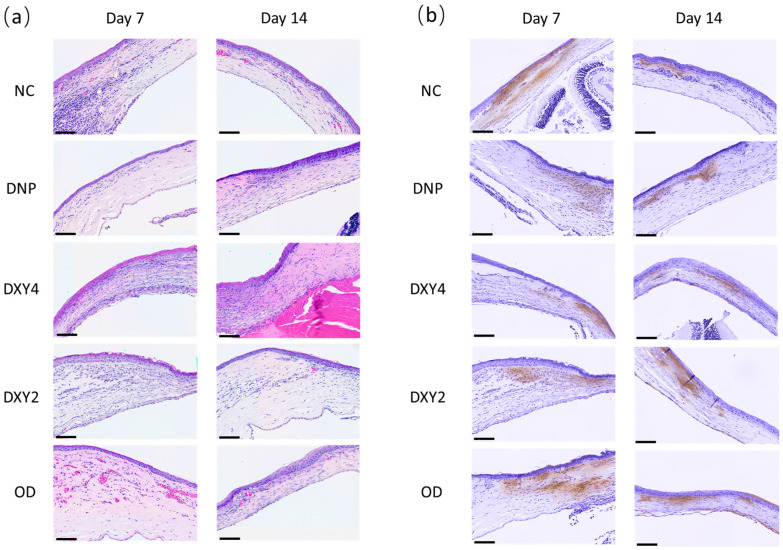
Neutrophil cell count and α-SMA staining by days 7 and 14. (**a**) HE staining of corneas indicates neutrophil cell infiltration in the stromal layer of the cornea. The DNP group showed the least infiltration on both days. Heavy cell counts were noted in the NC and DO groups. (**b**) α-SMA staining of the cornea. The DNP group show relatively minimal staining on both days 7 and 14. Scale bars: 100 µm.

The neutrophil counts on both day 7 and day 14 were significant between groups. (F (4, 11) = 33.588, *p* < 0.001) and (F (4, 8) = 65.779, *p* < 0.001), respectively (Figure 6 and Figure 8). On day 7, post hoc multiple comparisons showed that the NC and OD groups had significantly higher neutrophil infiltration than other three treatment groups. A post hoc Tukey’s HSD test on day 7 was reported comparing the NC to the DNP group, *p* < 0.001, 95% C.I. = [258.760, 598.574]; DXY4 group, *p* < 0.001, 95% C.I. = [222.760, 562.574]; and the DXY2 group, *p* < 0.001, 95% C.I. = [150.426, 490.240]. Meanwhile, the OD group was also highly infiltrated with neutrophils as compared to the DNP group, *p* < 0.001, 95% C.I. = [250.233, 568.100]; the DXY4 group, *p* < 0.001, 95% C.I. = [214.233, 532.100]; and the DXY2 group, *p* < 0.001, 95% C.I. = [141.900, 459.767]. By day 14 of the study, a post hoc Tukey’s HSD test compared the NC to the DNP group, *p* < 0.001, 95% C.I. = [103.483, 221.850]; DXY4 group, *p* < 0.001, 95% C.I. = [103.483, 221.850]; and DXY2 group, *p* < 0.001, 95% C.I. = [102.998, 235.336]. Meanwhile, the OD group also had significantly higher neutrophil infiltration than the DNP group, *p* < 0.001, 95% C.I. = [160.331, 292.670]; DXY4 group, *p* < 0.001, 95% C.I. = [160.331, 292.670]; and DXY2 group, *p* < 0.001, 95% C.I. = [160,515, 305.485].

#### 3.3.2. Effects of Doxycycline on Myofibroblasts

After the first week of treatment, the NC and OD groups exhibited the highest α-SMA expression (Figure 6 and Figure 8). DXY4 maintained the lowest expression among the five groups in both the 7- and 14-day studies ((F (4, 11) = 29.637, *p* < 0.001) and (F (4, 8) = 9.164, *p* = 0.004), respectively). Both NC and OD groups had significantly higher mean scores than the 3 topical groups. With post hoc Tukey’s HSD test reporting the NC group higher than the DNP group (*p* < 0.00, 95% C.I. = [0.2, 0.527]); the DXY4 group (*p* < 0.001, 95% C.I. = [0.225, 0.551]) and the DXY2 group (*p* = 0.003, 95% C.I. = [0.086, 0.412]). While the OD group was scored higher than the DNP group (*p* < 0.001, 95% C.I. = [0.199, 0.504]); the DXY4 group (*p* < 0.001, 95% C.I. = [0.223, 0.528]); and the DXY2 group (*p* = 0.003, 95% C.I. = [0.084, 0.39]). After two weeks of treatment, OD showed a constantly high MF expression. Only the NC group had less alpha-SMA staining when a longer treatment was applied. A post hoc Tukey’s HSD test demonstrated that the DXY4 group had significantly less α-SMA IRS than the DXY2 and OD group (*p* = 0.011, 95% C.I. = [−0.404, −0.056]); and (*p* = 0.004, 95% C.I. = [−0.443, −0.096], respectively).

## 4. Discussion

The synthesis of DNPs fit the characteristics of nanoparticles that exhibited therapeutic effects in our rat model. Since our target organ was the cornea, drug delivery to this target organ was the least of the challenges. Due to the anatomy of the nasolacrimal ducts and physiological blinking, the improvement of drug absorption and loading then sustained at the target organ was the goal we tried to achieve in this study. This DNP preparation had a small particle size of 132.6 ± 0.2 d.nm with a high positive zeta potential of 22.4 ± 1.01 mV. The corneal epithelium is negatively charged at physiological pH. This negative charge of mucin coating (glycosyl amino glycans lining) on the corneal epithelium surface can be exploited to improve the residence time of the formulation on the precorneal surface [34,35]. Our DNP eyedrop preparation was cationic. Thus, an electrostatic attraction towards the corneal surface will prolong the residence time of the formulation.

In this study, we successfully induced deep corneal wounds in rats by performing incisional lamellar keratectomy. Liu et al. mentioned several animal models that have been used to study corneal defects, including rabbits, mice, rats, zebrafish, pigs, and chickens [36]. Several approaches have been used to create corneal stromal wounds, such as ring drill trephination into the central cornea in mice and then separation of its epithelium and basement membrane [37], photorefractive keratectomy in rabbits [38,39], and incisional wounds using a surgical blade to penetrate the cornea and create defects in mice and rabbits [40,41]. In our case, rats were more feasible and easier to handle; a biopsy punch was used to mark the wound edge and incisional keratectomy was performed to create a deep stromal wound. The model utilized SD rats that had average cornea thickness of about 150 µm. This microsurgery has to be performed with precision and speed (not more than 10 min) in order to produce wounds of the same thickness with minimal stromal edema during the OCT imaging immediately post-surgery. The healing process took place immediately after injury, allowing us to observe the changes in clinical presentation and postmortem histopathological appearance.

The epithelial healing underwent three phases: the lag phase, the cell migration and reepithelization phase, and then the cell proliferation, stratification, and differentiation phase [42,43]. Immediately after injury, the adjacent unwounded epithelial cells flattened and migrated to the denuded surface to seal the wound [44,45]. There was no difference in the fluorescein-stained area (epithelial healing rate) among our control and treatment groups. This animal model was surgically induced with minimal expectations of complications during the healing process; hence, it is acceptable, as the rate of restoration of surface epithelial integrity was uneventful [3]. The usage of doxycycline in our case does not directly enhance the lag phase, cell migration phase, or corneal reepithelization phase clinically. However, the later phases were speculated to have been affected. During the day 7 observation, the NC group and OD group exhibited more layers of hyperplastic cuboidal epithelial basal cells, contributing to significant epithelial thickening. Corneal epithelium wounds demonstrated limbal stem cell (LESC) proliferation within 24 h after wounding. Pro-inflammatory cytokines and growth factors modulate the LESC upregulation [46,47]. We observed that some rats in the DXY4 and DXY2 groups had mitotic figures at the basal cell layer (Figure 5d,e) by day 7, while the OD and NC exhibited haphazardly proliferated basal cell layers with defective basement membrane, suggesting that individuals without treatment may have prolonged or delayed epithelial cells proliferation phase. Upregulation and activation of MMP-9 during injury caused ECM degradation and modified cellular adhesive properties, hence hindering the formation of desmosomes in the cell-to-cell junctions at the basal cell layer [48,49]. Another study also showed that corticosteroid and doxycycline suppressed MMP-9, MAPK activation in the epithelium in a dry eye model [50]. The application of DNP eye drops apparently shortens the period of epithelial proliferation and progress into differentiated basal cells, wing cells, and stratified keratinocytes.

Numerous studies have demonstrated that many growth factors, including vascular endothelial cell growth factor (VEGF), basic fibroblast growth factor (FGF-2), and tumor necrosis factor-alpha (TNFα) regulate angiogenesis and also have a cross-effect on MMP expression [6,51,52]. A previous study has shown that culturing endothelial cells would form capillary-like tubules that produce MMPs 2, 9 and MT-1 (membrane type-1) MMP, and then these tubules’ formation would be disrupted when given MMP inhibitors [53]. Topical application of doxycycline eye drops (regardless of their formulation) showed a pattern hindering corneal neovascularization throughout the study. The gel nanoparticle form of doxycycline did show effects immediately post-surgery with a minimal initial CNV and continuous reduction in blood vessels on the cornea. Rats who received topical doxycycline had a relatively high initial CNV after regular treatment, and then they started a reduction by day 7. The DNP eyedrops successfully exhibited better potency in their antiangiogenic effects in our study. They showed superior effects and earlier therapeutic onset compared to other forms of doxycycline treatment. We speculated that the particle size and its positive zeta potential allowed the medication to bind to the cornea for a longer time and provide better absorption, though the concentration of doxycycline in the DNP was 10 times lower and administered only two times per day.

The expression of MMP-2 and MMP-9 in our study was analyzed with immunohistochemistry of the cornea on days 7 and 14. The NC group had the highest reactivity score on both days, indicating ongoing inflammatory cytokine proliferation and upregulation of MMPs during these times. In a study with cultured human corneal epithelium stimulated with lipopolysaccharide or TGF-β1, doxycycline was able to inhibit IL-1α mRNA expression [13] and TGF-β1-induced MMP-9 via Smad and MAPK signaling pathway [54], respectively. Our study showed that doxycycline treatment, regardless of route, formulation, or dosage, significantly reduced MMP-2 and MMP-9 immunoreactivity scores by day 7. The DNP group exhibited a better suppression effect on MMP-2 and MMP-9 expression throughout the study. We speculated that the binding of nanosized doxycycline solution to the activated MMPs was better compared to other forms of doxycycline.

Neutrophil cell counts were performed on H&E-stained histopathology slide. The neutrophils were identified and counted as cells with multilobulated basophilic nucleus and eosinophilic cytoplasm [55]. This is no doubt a limitation in this study. In future research, the application of IHC using different antibodies such as CD66b, Myeloperoxidase(MPO), and Elastase [56] would have be more specific in highlighting the immunoreactivity of the neutrophils

Statistically, the NC and DXY4 groups were significantly thicker than a normal corneal stromal layer. The initial thickening was due to the edema and inflammation phase which begin immediately after injury. Subsequently, high proliferation and then the presence of myofibroblast complemented the misarranged extracellular matrix sequel. The development and maturation of the myofibroblast from its progenitors (the corneal fibroblast cells and bone marrow-derived fibrocytes) occurred after cornea injuries or infections that involved the epithelium basement membrane (EBM) [57,58]. The ongoing supply of adequate TGF-β and PDGF is essential for the development of mature myofibroblasts from these precursor cells and persistence in the stroma. In superficial stromal wounds, the EBM is fully regenerated within 8 to 10 days, thus cutting off the supply of epithelium-derived TGFβ and PDGF. Severe loss of epithelial and stromal cells that upregulate MMP expression prolonged the healing time of EB, giving way to the maturation of myofibroblasts in the stroma resulting in fibrotic scarring of the cornea [59,60,61]. α-SMA positive cell count on day 14 was higher than day 7 for all the treatment groups that received doxycycline. The control group had an opposite expression trend; though cell count by day 14 was less than day 7, its total count on day 14 still resembled the treated groups. Mohan et. al. 2003 demonstrated a similar scenario where deep PRK resulted in an upregulation of SMA-positive cells by week 1 [62]. Depending on the size and depth of the stromal wound, the remodeling phase occurred 2–3 weeks up to 2 months in rats and rabbits. The corneal fibroblasts and the fibrocytes that migrate into the corneal stroma begin their developmental program into mature α-SMA positive myofibroblasts during this 2–3 week [58,62]. In the DNP group, α-SMA IRS were slightly higher by day 14, our observation of the NC group was only 14 days, suggesting a possibility of delayed fibrosis. The myofibroblast expression was lower by day 14, but the score was still similar to the DNP group, the stromal thickness was very high throughout 14 days, suggesting that the contraction and remodeling had not started.

In the context of the corneal wound healing model, another animal model, the sulfur mustard wounding model in murine or rabbits was more widely used in recent years. This model exhibits 2 phases of healing where the initial response was a severe injury to the epithelium and possible anterior stroma layer with massive necrosis. Our model provides a more direct perspective of corneal wound healing that involves the deep stromal layer. The excision of a large quantity of stromal tissues and epithelium allow us to induce a moderate inflammatory keratitis; this was an ideal condition to see the difference during treatments of doxycyclines.

## 5. Conclusions

This doxycycline-loaded gel nanoparticle preparation had a small particle size of 132.6 ± 0.2 d.nm with a high positive zeta potential of 22.4 ± 1.01 mV. This DNP demonstrated a sustained-release profile, enhanced cellular uptake efficiency, and prolonged retention time within the cornea. Compared to the regular doxycycline eyedrop in DXY4 and DXY2, the dosage of doxycycline in nanoparticle form was 10 times less, and the application frequency was as good as the minimum requirement (DXY2) among all topical treatment groups. In our wound model, DNP effectively reduced pathological neovascularization, expression of MMP-2 and MMP-9, and neutrophil infiltration throughout the study. It also regulated the myofibroblast expression which indirectly showed a minimally thickened epithelial and stromal layer during the study. In conclusion, our findings highlighted the therapeutic potential of DNP in enhancing the deep corneal wound healing process. This indicates a good potential for clinical applications in veterinary and comparative medicine.

## Figures and Tables

**Table 1:** Different Dxy groups and their tested conditions in a rat model of deep corneal wound.

Groups	NC	OD	DXY2	DXY4	DNP
Dxy concentration	/	10 mg/kg	0.1%	0.1%	0.01%
Dosing times (day)	/	2	2	4	2

**Table 2:** Characterization of GNP with/without Dxy loading.

	Size (nm)	Zeta (mV)	PdI	E.E. (%)
GNP	98.83 ± 0.68	21.5 ± 0.30	0.117 ± 0.033	/
DNP	132.0 ± 0.20	22.4 ± 1.01	0.143 ± 0.007	53.80% ± 4.25

Data presented as mean ± SD, *N* = 4.

## Data Availability

The data presented in this study are available on request from the corresponding author.

## Supplementary Materials

The following supporting information can be downloaded at: https://www.mdpi.com/article/10.3390/vetsci12020143/s1, Figure S1. Measurement of blood vessels on the cornea; Figure S2. Corneal epithelial and stromal thickness measurement via OCT images.
