# Supplementary material for: Therapeutic Assessment of Diverse Doxycycline-Based Formulations in Promoting Deep Corneal Wound Healing: Evidence from a Rat Model
Source: Vet Sci. 2025 Feb 8;12(2):143. doi: 10.3390/vetsci12020143 (PMC11860526; doi:10.3390/vetsci12020143)
Supplement: Supplementary file 1 [file vetsci-12-00143-s001.zip › vetsci-3405005-supplementary.pdf]

Supplementary Material

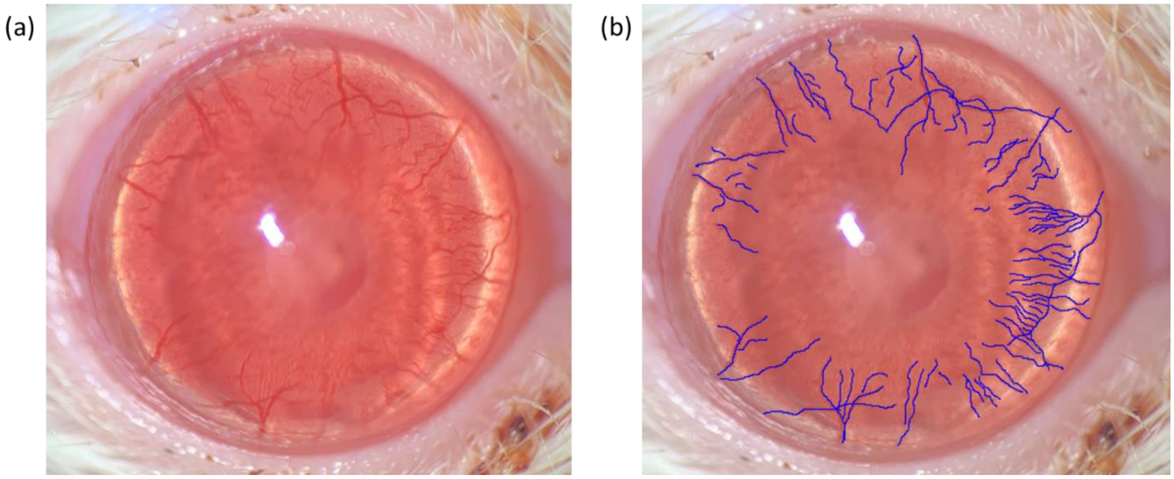

Figure S1. Measurement of blood vessels on the cornea. (a) The original photo of a rat. (b) Tracing of the blood vessels using ImageJ software 1.38x (National Institutes of Health, Bethesda, Maryland, USA). Summation of the total length of blood vessels traced were recorded.

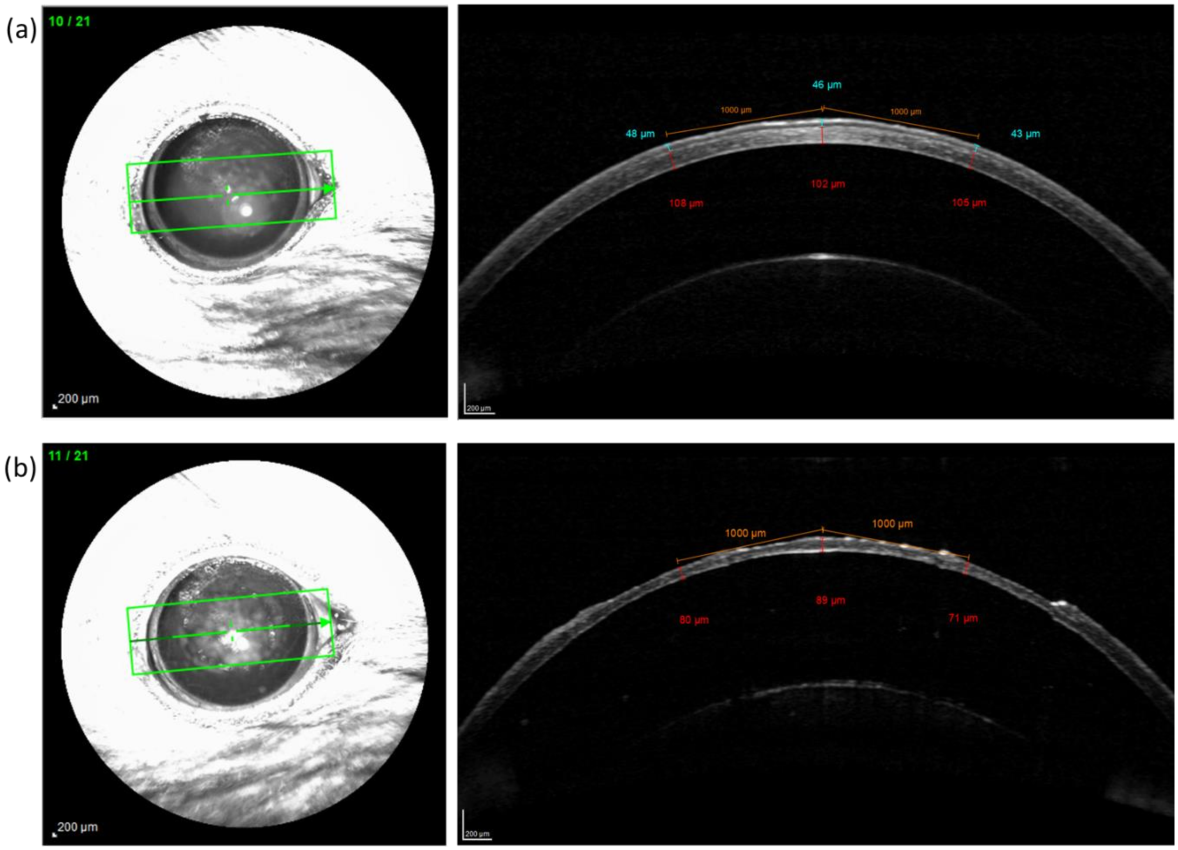

Figure S2. Corneal epithelial and stromal thickness measurement via OCT images. (a) A representative image of a normal cornea with epithelium and stromal intact. (b) A representative image of a post-surgery Day-0 (immediately after surgery) cornea. Measurements were taken at the central point of the cornea, and 1000 μm medial and lateral side from the central point. Measurements of the thickness was calculated as mean of these 3 measurement points.
